# Depression, anxiety, fatigue, and quality of life in a large sample of patients suffering from head and neck cancer in comparison with the general population

**DOI:** 10.1186/s12885-020-07773-6

**Published:** 2021-01-22

**Authors:** C. Hammermüller, A. Hinz, A. Dietz, G. Wichmann, M. Pirlich, T. Berger, K. Zimmermann, T. Neumuth, A. Mehnert-Theuerkauf, S. Wiegand, V. Zebralla

**Affiliations:** 1 Clinic of Otolaryngology, Head and Neck Surgery, University of Leipzig, Leipzig, Germany; 2 Department of Medical Psychology and Medical Sociology, University of Leipzig, Leipzig, Germany; 3 Innovation Center Computer Assisted Surgery (ICCAS), University of Leipzig, Leipzig, Germany

**Keywords:** Head neck cancer (HNC), Survivorship, Depression, Aftercare, Fatigue, Quality of life (QOL)

## Abstract

**Background:**

Treatment of head and neck cancer (HNC) often leads to visible and severe functional impairments. In addition, patients often suffer from a variety of psychosocial problems, significantly associated with a decreased quality of life. We aimed to compare depression, anxiety, fatigue and quality of life (QoL) between HNC patients and a large sample of the general population in Germany and to examine the impact of sociodemographic, behavioral and clinical factors on these symptoms.

**Methods:**

We assessed data of HNC patients during the aftercare consultation at the Leipzig University Medical Center with a patient reported outcome (PRO) tool named “OncoFunction”. Depression, anxiety, fatigue and QoL were assessed using validated outcome measures including the PHQ-9, the GAD-2, and the EORTC QLQ-C30 questionnaire.

**Results:**

A total of 817 HNC patients were included in our study and compared to a sample of 5018 individuals of the general German population. HNC patients showed significantly higher levels of impairment in all dimensions assessed. Examination of association between depression, anxiety, fatigue and QoL and clinical as well as sociodemographic variables showed significant relationships between occupational status, ECOG-state, body mass index and time since diagnosis.

**Conclusions:**

HNC patients suffer significantly from psychological distress. The used questionnaires are suitable for the use in daily routine practice and can be helpful to increase the detection of depression, anxiety and fatigue and therefore can improve HNC aftercare.

## Background

Patients treated for head and neck cancer (HNC) have a high risk for loss of function such as swallowing, eating, and speaking, and therapy-associated side effects. Anxiety, depression, and fatigue are frequent psychological symptoms and syndromes particularly among HNC patients [1–4]. The American Head and Neck Survivorship Care Guideline defined the detection of negative psychological side effects as one major target of the tumor aftercare in addition to functional assessments and physical examinations for early detection of recurrence or secondary primary tumor [5]. The visibility of HNC itself and the impairment through its therapy often negatively impact psychological status such as depression and anxiety. Moreover, it is known that there is a high risk of underreporting psychosocial issues in HNC patients [6]. Research suggests that obtaining patient reported outcome (PRO) in a structured way using validated questionnaires could be an adequate way to overcome this problem. PRO is defined as any report of patient’s health that comes directly from the patient [7]. The lack of valid assessment of psychosocial issues such as depression and anxiety was linked to a higher mortality and a significantly reduced quality of life (QoL) [8–10].

Depression is a common mental disorder in HNC patients [11, 12]. There is a wide range of prevalence of depressive symptoms in HNC patients after radiotherapy (29–42%) [13], while other data reported a prevalence of only 6% [14]. The prevalence also depends on time after diagnosis [15]. Fear of cancer recurrence is a common anxiety in cancer patients. Moreover, aspects of anxiety associated with loss of occupation, social isolation or social status play an important role [16, 17]. Fatigue is also a common symptom of cancer patients. Bossi et al. reported an incidence of moderate to severe fatigue in 18% of their HNC patients [2]. Fatigue impairs all aspects of life, but it is also hardly comprehensible and is not easy to treat.

Functional impairments like speaking, swallowing and eating problems as well as aesthetic changes lead to a loss of social contact and decreased QoL in HNC patients after treatment [18, 19]. Moreover, a high rate of post-traumatic distress in HNC survivors has been shown [20].

The objective of this study was to compare depression, anxiety, fatigue and QoL in HNC patients with a large sample of the general population in Germany and to examine the impact of sociodemographic, behavioral and clinical factors on these symptoms and elucidate the relationship among these variables.

## Methods

### HNC patients

**Fig. 1:**
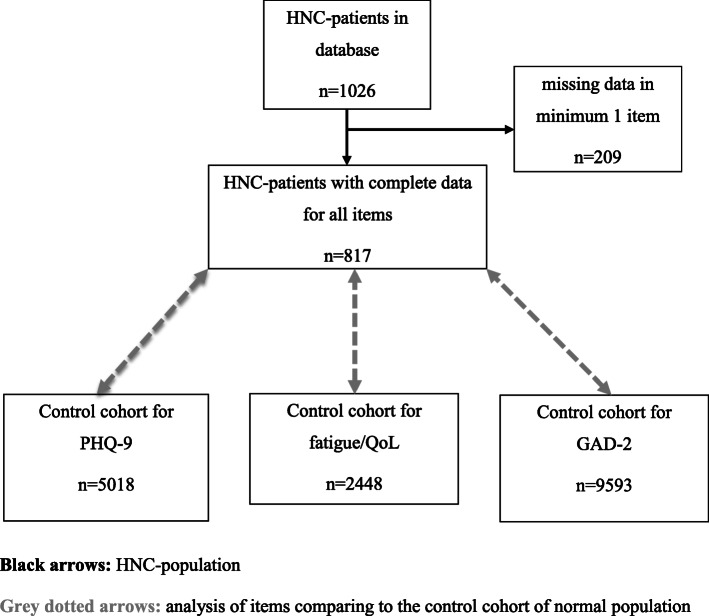
CONSORT diagram demonstrating case selection in the head and neck cancer group and the normal population

All patients of the HNC sample were participants of the aftercare and survivorship program of the University Medical Clinic of Otolaryngology, Head and Neck Surgery. Since 2013 we established our program called “OncoFunction”, an electronic patient reported outcome measurement (ePROM) based on the International Classification on Functioning (ICF) [7, 21]. Depending on the ICF, tools were defined and included in the questionnaire, which are recommended by the German Cancer Society [22]. The aftercare schedule is based on the recommendations of the NCCN guidelines for HNC follow-up. Every patient consulting our aftercare consultation completed the questionnaire via tablet computer. The collected data are visualized for the physician to ease detection of problems in various dimensions in their kinetic. The usability of PRO and the successful implementation of OncoFunction in the daily routine had been shown in previous studies [23, 24]. At the time of the database lock for the present analyses, 1026 HNC patients had a minimum of one consultation in our aftercare and were included (Fig. 1). We used only the first PRO of every patient for our analysis, before any intervention could influence the following results. Only patients who were treated with curative intent were included.

### Samples of the general population

#### PHQ-9

The data basis for the control group concerning the PHQ-9 was a survey of the German general population (*n* = 5018) [25]. Age, gender, and regional distribution were the major criteria for representativeness. The PHQ-9 item mean scores of the general population were taken from the publication of Hinz et al. [26].

#### GAD-2

For this questionnaire we used data from the LIFE-Adult Study, a German general population study with 9593 participants [27].

#### EORTC QLQ-C30 fatigue and QoL

The comparison group for the two scales of the EORTC QLQ-C30, fatigue and global health/QoL, was taken from a German general population study, which is sample German II in that publication [28]. This examination comprised 2448 participants from the German general population.

### Instruments

The used questionnaires are previously published and evaluated instruments.

#### PHQ-9

The PHQ-9 is a screening instrument with 9 items (see Table 2), developed to measure depressive symptoms. For each item the patients were asked to assess how much they were bothered by the symptoms over the last 2 weeks. There were four answer options: not at all (0), several days (1), more than half of the days (2), and nearly every day (3). The sum score (range 0 to 27) indicate the degree of depression, with scores of ≥5, ≥10, and ≥15 representing mild, moderate, and severe levels of depression [29].

#### GAD-2

The Generalized Anxiety Disorder Questionnaire GAD-2 is a 2-item short form of the GAD-7 [30]. The answer options were equal to those of the PHQ-9, resulting in a score range from 0 to 6. Normative values are available [27].

#### EORTC QLQ-C30 fatigue and QoL

This questionnaire [31] was developed to assess QoL in cancer patients. It comprises five functioning scales (physical, role, cognitive, emotional, and social functioning), three symptom scales, six single items and one 2-item global health status / QoL scale. In this study, only the 2-item global quality of life scale (called QoL scale) and the three-item fatigue scale were used. All scales of the questionnaire were transformed to the range from 0 to 100. Higher scores in fatigue indicate more severe problems, whereas higher scores in the QoL scale represent better QoL. Normative values for this questionnaire are available [28].

#### Other variables

Additionally, the ECOG (Eastern Cooperative Oncology Group) state, behavioral items (smoking and consumption of alcohol), the occupational state and Body Mass Index (BMI) were assessed at every aftercare consultation.

### Statistical analysis

The general populations mean scores for the subsamples defined by gender and age group (≤ 64 years vs. ≥ 65 years) were calculated as follows:

For the EORTC QLQ-C30 and for the GAD-2 we had access to the original data and calculated the mean scores accordingly. For the PHQ-9 we used the mean scores of the normative study [25]. From the mean scores which are given there in terms of age decades we calculated weighted means for the subgroups < 65 y and ≥ 65 y), separately for males and females.

The total mean scores of the control groups for comparison on mean score level (Table 2) were calculated as the weighted means of the four groups (gender * age group). The proportions of the four groups in the HNC group were 58.5% (males < 65 y), 41.5% (males ≥ 65 y), 57.5% (females, < 65 y), and 42.5% (females, ≥ 65 y). These percentages were used to weight the means of the four groups in Table 2 and to calculate the weighted percentages of the depression cases in the general population.

Age and gender effects on the dependent variables (depression, anxiety, fatigue, and QoL) were tested with two-way analyses of variance (ANOVAs). For the PHQ-9 part-whole-corrected item-test-correlations and Cronbach’s alpha for measuring internal consistency were calculated. The one-dimensional structure of the PHQ-9 was tested with a confirmatory factor analysis (CFA) using the following coefficients: Chi^2^, comparative fit index (CFI), Tucker-Lewis index (TLI), root mean square error of approximation (RMSEA), and standardized root mean square residual (SRMR).

Associations between (behavioral and clinical) prognostic factors and the dependent variables were statistically tested with analyses of variance (ANOVAs). In these ANOVAs, age and sex were included as covariates.

Associations among the dependent variables were expressed with Pearson correlations. All statistical calculations were done using SPSS version 20, with the exception of the CFA that was calculated with MPlus.

## Results

### Sample characteristics

The original sample comprised 1026 patients (Fig. 1). The four scales PHQ-9, GAD-2, EORTC QLQ-C30 fatigue and EORTC QLQ-C30 QoL were completed by 854, 869, 869, and 868 participants, respectively. In this analysis we used the data of those patients who completed all of these four scales (*n* = 817). The sample consisted of 631 males (77.2%) and 186 females (22.8%), the mean age of the 817 patients was 62.7 years (SD = 10.4 years). Table 1 shows the characteristics of this sample.

**Table 1:** Characteristics of the sample of H&N cancer patients

	Total (*n*=817)		Males (*n*=631)		Females (*n*=186)	
	N	%	N	%	N	%
Age group						
18–64 y.	476	58.3	369	58.5	107	57.5
≥ 65 y.	341	41.7	262	41.5	79	42.5
Occupation^a^						
Not occupied	625	76.5	490	77.7	135	72.6
Occupied	192	23.5	141	22.3	51	27.4
Alcohol drinking						
No	602	73.7	435	68.9	167	89.8
Yes	215	26.3	196	31.1	19	10.2
Smoking						
No	601	73.6	446	70.7	155	83.3
Yes	216	26.4	185	29.3	31	16.7
Tumor group						
Oral cavity	124	15.2	88	13.9	36	19.4
Oropharynx	285	34.9	220	34.9	65	34.9
Larynx, Hypopharynx	246	30.1	220	34.9	26	14.0
Other	162	19.8	103	16.3	59	31.7
Tumor stage^a^						
I	141	19.2	105	18.2	36	22.5
II	102	13.9	82	14.2	20	12.5
III	122	16.6	89	15.5	33	20.6
IV	371	50.4	300	52.1	71	44.4
Treatment group						
1: OP + RT - CT-	215	26.3	159	25.2	56	30.1
2: OP + RT + CT-	199	24.4	159	25.2	40	21.5
3: OP + RT + CT+	246	30.1	192	30.4	54	29.0
4: OP - RT + CT+	116	14.2	93	14.7	23	12.4
5: Other	41	5.0	28	4.4	13	7.0
Metastases						
No	441	54.0	337	53.4	104	55.9
Yes	376	46.0	294	46.6	82	44.1
ECOG performance^a^						
0	205	34.4	157	34.2	48	35.0
1	319	53.5	249	54.2	70	51.1
2–4	72	12.1	53	11.5	19	13.9
Body Mass Index						
< 20 kg/m^2^	135	16.5	89	14.1	46	24.7
20 – < 25 kg/m^2^	391	47.9	314	49.8	77	41.4
25 – < 30 kg/m^2^	216	26.4	176	27.9	40	21.5
≥ 30 kg/m^2^	75	9.2	52	8.2	23	12.4
Time since diagnosis^a^						
≤ 9 months	429	52.6	324	51.4	105	56.8
> 9 months	386	47.4	306	48.6	80	43.2

*OP* Surgery, *RT* Radiotherapy, *CT* Chemotherapy

^a^Missing data not reported

### Mean score comparisons between the HNC patients and the general population

**Fig. 2:**
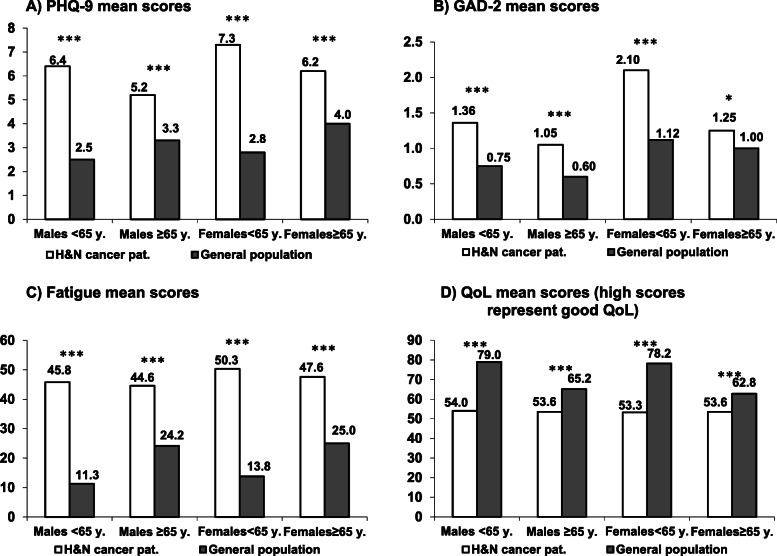
Mean scores of the dependent variables, broken down by gender and age group, for **a** PHQ-9; **b** GAD-2; **c** Fatigue; **d** QoL. *: *p*<.05; ***: *p*<.001

Figure 2 shows the mean scores of the HNC patients and the general population comparison group, broken down by gender and age group. The burden of the HNC patients was significantly higher than that of the general population. In all four variables, the difference between the HNC patients and the general population was higher in the younger age group compared to the older age group.

### ANOVA results for age and gender differences (patients only)

Within the HNC group, the ANOVA gender and age effects were as follows: PHQ-9: gender: (*F* = 3.82, *p* = 0.051), age group (*F* = 5.40, *p* = 0.020), and gender * age group (*F* = 0.01, *p* = 0.946); GAD-2: gender (*F* = 12.6, *p* < 0.001), age group (*F* = 13.6, *p* < 0.001), and gender * age group (*F* = 0.065, *p* = 0.798); fatigue: gender (*F* = 2.10, *p* = 0.147), age group (*F* = 0.856, *p* = 0.355), and gender * age group (*F* = 0.246, *p* = 0.620), and QoL: gender (*F* = 0.029, *p* = 0.864), age group (*F* = 0.502, *p* = 0.479), and gender * age group (*F* = 1.269, *p* = 0.260).

The HNC patients showed poorer values in all symptom scales (depression, anxiety, and fatigue) as well as a lower level of QoL when compared with the general population. The same was found for the PHQ-9 and for all of its items. The most pronounced difference was found for fatigue with an effect size greater than 1. Among the depression items, agitation/retardation (*d* = 0.64), appetite problems (*d* = 0.61), and sleep problems (*d* = 0.54) showed the greatest differences, while for the items self-blame, concentration problems, and suicidal ideations there were only marginal differences (*d* between 0.07 and 0.16).

According to the classification of the PHQ-9 scores proposed by the original test authors [29], the percentages of depression symptom classes were as follows: no depression (51.3%), mild depression (25.9%), moderate depression (12.0%, and severe depression (10.8%). The corresponding percentages of the general population sample were: no depression (76.0%), mild depression (17.7%), moderate depression (4.8%), and severe depression (1.5%).

### Psychometric properties of the scales

The right part of Table 2 shows coefficients of internal consistency (*Cronbach’s alpha*) for all scales and a detailed item analysis for the PHQ-9. The alpha coefficients of fatigue and QoL scales were both above 0.90, of PHQ-9 and GAD-2 0.89 and 0.87, respectively. Concerning the PHQ-9, all items positively contributed to the PHQ-9 total scores with item-test correlations of at least 0.50. The highest contribution was from item 2 (*feeling depressed*).

**Table 2:** Mean score comparisons between the HNC patients and the general population

Scale / Item	HNC patients		General population		*d*	*r*_it_ HNC
	*M*	*SD*	*M*	*SD*		
**PHQ-9**						
Item 1. Loss of interest	0.81	0.95	0.52	0.63	0.37	0.75
Item 2. Feeling depressed	0.67	0.90	0.38	0.60	0.39	0.79
Item 3. Sleep problems	1.13	1.11	0.63	0.75	0.54	0.59
Item 4. Loss of energy	1.08	1.01	0.65	0.70	0.50	0.77
Item 5. Appetite problems	0.77	1.03	0.28	0.56	0.61	0.62
Item 6. Self-blame	0.26	0.63	0.20	0.48	0.11	0.58
Item 7. Concentration problems	0.44	0.74	0.39	0.60	0.07	0.66
Item 8. Agitation/retardation	0.60	0.93	0.16	0.45	0.64	0.61
Item 9. Suicidal ideation	0.17	0.51	0.10	0.35	0.16	0.51
**PHQ-9 Sum score (range 0–27)**	**5.95**	**5.88**	**3.30**	**3.65**	**0.56**	α**=0.89**
95% CI of mean	[5.55–6.35]		[3.20–3.40]			
**GAD-2 (range 0–6)**	**1.35**	**1.70**	**0.77**	**1.15**	**0.41**	α**=0.87**
95% CI of mean	[1.23–1.47]		[0.75–0.79]			
**C30 Fatigue (range 0–100)**	**46.2**	**28.4**	**17.1**	**21.6**	**1.16**	α**=0.91**
95% CI of mean	[44.2–48.2]		[16.2–18.0]			
**C30 QoL (range 0–100)**	**53.8**	**21.9**	**73.0**	**19.6**	**−0.93**	**α=0.91**
95% CI of mean	[52.3–55.3]		[72.2–73.8]			

*d* Effect size for the comparison H&N patients – general population, *r*_*it*_ Part-whole corrected item-test correlation, *α* Cronbach’s alpha

The CFA results of the one-dimensional PHQ-9 model resulted in the following coefficients:

- Chi^2^ (DF) = 319.264 (27), CFI = 0.918, TLI = 0.891, RMSEA = 0.114, and SRMR = 0.049.

### Associations between sociodemographic, behavioral, and clinical factors and depression, anxiety, fatigue, and QoL

Table 3 presents the mean symptom scores for subgroups of the sample defined by sociodemographic, behavioral, and clinical variables.

**Table 3:** Mean scores depending on sociodemographic and clinical variables

	*n*	PHQ-9		GAD-2		Fatigue		QoL	
		M	SD	M	SD	M	SD	M	SD
Occupational status		***p*<.001**		*p*=.110		***p*<.001**		***p*<.001**	
Not occupied	625	6.2	6.0	1.34	1.71	47.8	28.5	52.0	21.6
Occupied	192	5.0	5.3	1.38	1.67	40.7	27.4	59.5	21.8
Alcohol drinking		*p*=.086		*p*=.139		***p*<.001**		*p*=.442	
No	602	6.1	5.9	1.40	1.72	48.3	28.4	53.4	21.4
Yes	215	5.4	5.7	1.20	1.64	40.0	27.7	54.8	21.3
Smoking		*p*=.153		*p*=.392		*p*=.319		*p*=.678	
No	601	5.7	5.7	1.29	1.66	46.7	28.5	53.9	22.2
Yes	216	6.6	6.3	1.50	1.80	44.8	28.1	53.3	21.1
Tumor group		*p*=.304		*p*=.943		*p*=.275		*p*=.890	
Oral cavity	124	6.3	5.6	1.40	1.70	44.3	28.3	53.2	21.1
Oropharynx	285	6.3	5.8	1.38	1.75	48.5	27.4	53.2	21.6
Larynx, Hypopharynx	246	5.9	6.2	1.29	1.68	45.6	29.0	54.2	23.0
Other	162	6.0	5.9	1.35	1.70	46.2	28.4	53.8	21.9
Tumor stage		*p*=.489		*p*=.494		*p*=.067		***p*=.012**	
I	141	5.5	6.1	1.31	1.68	40.9	27.9	58.0	22.5
II	102	5.5	5.7	1.04	1.40	45.0	29.2	55.4	22.1
III	122	6.4	6.2	1.47	1.94	46.0	30.7	54.7	24.7
IV	371	6.2	5.9	1.34	1.65	48.1	27.0	51.3	20.5
Treatment group		*p*=.341		*p*=.523		***p*=.015**		*p*=.213	
1: OP + RT - CT-	215	5.5	5.9	1.27	1.67	41.4	28.2	55.8	21.7
2: OP + RT + CT-	199	5.5	5.6	1.23	1.67	46.0	28.9	54.6	22.3
3: OP + RT + CT+	246	6.5	5.8	1.53	1.76	48.3	27.4	51.6	20.5
4: OP - RT + CT+	116	6.1	6.1	1.29	1.63	47.3	28.4	54.2	24.3
5: Other	41	6.7	6.5	1.41	1.95	55.6	30.2	50.2	20.8
Metastases		*p*=.087		*p*=.430		*p*=.054		*p*=.356	
No	441	5.6	5.9	1.29	1.71	44.4	28.4	54.4	22.8
Yes	376	6.4	5.9	1.41	1.70	48.3	28.3	53.0	20.8
ECOG performance		***p*<.001**		***p*<.001**		***p*<.001**		***p*<.001**	
0	205	3.5	4.4	0.89	1.34	33.8	25.2	62.2	22.0
1	319	6.5	5.8	1.40	1.61	49.1	26.6	51.0	20.3
2–4	72	9.8	7.3	2.03	2.16	70.8	23.8	41.8	20.8
Body Mass Index		***p*=.049**		*p*=.0534		***p*=.002**		***p*<.001**	
≤ 20 kg/m^2^	135	7.0	6.0	1.52	1.81	52.5	29.4	47.6	22.8
20 – ≤ 25 kg/m^2^	391	6.2	6.1	1.39	1.76	47.4	28.0	53.6	21.5
25 – ≤ 30 kg/m^2^	216	5.0	5.5	1.16	1.51	40.4	28.3	55.6	21.8
> 30 kg/m^2^	75	5.7	5.5	1.35	1.72	45.0	26.3	60.1	20.2
Time since diagnosis		***p*<.001**		***p*=.015**		***p*=.001**		***p*<.001**	
≤ 9 months	429	6.7	6.1	1.50	1.81	49.4	28.1	51.1	20.8
> 9 months	386	5.1	5.5	1.18	1.57	42.6	28.4	56.7	22.7

*OP* Surgery, *RT* Radiotherapy, *CT* Chemotherapy, *p value* Significance of the ANOVA with age group and gender as covariates

Occupied patients achieved significantly lower values in PHQ-9 and fatigue and had better QoL scores. Patients continuing alcohol and tobacco consumption had no significant different results regarding all scores but fatigue, that self-reportedly was better in patients who continued alcohol intake (*p*< 0.001).

Tumor localization, metastases and treatment showed only a slight difference between groups, mostly without reaching significance. In tendency, patients with advanced tumor had higher PHQ-9, GAD-2 and fatigue scores. Only QoL was significantly better in patients with lower tumor stages whereas patients with unusual or trimodal treatment reported significantly higher values in fatigue.

Regarding ECOG performance scale all parameters showed a highly significant difference with greater impairment of patients with higher ECOG state. BMI was also significantly associated with depression, fatigue and QoL. The highest values in depression, anxiety and fatigue were reported from patients with a BMI< 20 kg/m^2^ whereas patients with a BMI > 30 kg/m^2^ reported best QoL. Patients with time since diagnosis longer than 9 months reported in general better QoL and lower scores in the psychometric scales.

## Discussion

We found patients with HNC generally having poorer values for PHQ-9, GAD-2, fatigue and QoL in comparison to the general population.

In our sample of 817 patients there were more male than female patients which is typical for a HNC patient cohort [32]. There are many patients below the age of 65 years (58.5%). The increasing number of human papillomavirus (HPV)-driven oropharyngeal carcinomas in younger patients emphasizes the importance of recording these parameters, especially in this group. Younger patients, especially females, were more affected by depression and anxiety (Fig. 2). The disease itself and its negative impact on working life, family status and planning the future may explain these negative findings [33].

Most of HNC patients (76.5%) were not occupied at the examination date. Unemployed patients achieved significantly higher values in PHQ-9 and fatigue scale and lower values concerning QoL while presence of anxiety was not significantly dependent on occupational state. Advanced HNC destroy tissue and organ functions, and even successful HNC treatment often triggers development of scars and fibrosis, which are leading to severe functional impairments regarding e.g. speaking and swallowing problems. This reduces the possibility of return to work significantly [34] and enormously compromises QoL. So far, it remains unclear if the occupational status protects from depressive symptoms, or alternatively, patients not being depression prone more often return to their job.

Approximately 26% of our patients continued alcohol drinking and smoking after diagnosis. We could not find a significant influence on PHQ-9, GAD-2 and QoL values. But surprisingly continued alcohol drinking was correlated with lower values in fatigue scale. A reason could be a modified perception of problems in patients with addictive disorders or alternatively freedom from pain and discomfort allowing a continued alcohol intake.

The most frequent tumor localization in our cohort was the oropharynx, followed by larynx and hypopharynx cancer. There were no signs for any differences between the entities respective to the localization of the primary lesion concerning PRO values in all scales. This highlights that being HNC patient alone can predict a risk of developing psychological comorbidities independent from the primary tumor’s site. As expected, patients with smaller tumors had a significantly better QoL. The absence of functional problems at time of diagnosis and the limited post-treatment impairment after organ and organ-function preserving therapy of smaller tumors as well as the perception of the disease rather as curable than as a life-threatening disease together with the reduced time-span needed to cure the cancer might explain these differences.

Considering the applied therapy, patients were separated in three criteria (surgery=OP, radiotherapy=RT, chemotherapy=CT). We found significant worse values in fatigue score in patients undergoing trimodal treatment. A possible reason can be a higher rate of therapy-related side effects by adjuvant radio- and chemotherapy in comparison to single surgery. Data of Moubayed et al. reported radiotherapy as a risk for developing fatigue and advanced tumor as a risk for impaired QoL and depression, respectively [35]. Moreover, Eyob et al. reported that also chemotherapy is predictive to the development of fatigue [36].

Concerning the presence of locoregional metastases, we did not find any significant differences in PHQ-9, GAD and QoL, but we saw a tendency in the fatigue scale for worse values in patients having locoregional metastases. Having metastasis mostly indicates a disease in an advanced stage. These findings were correlating to our data concerning tumor size.

Obviously, a better general health classified according to the ECOG to be of state 0 or 1 (good performance status) was linked to better values in all scales. These patients can better get back to their premorbid lifestyle, can perhaps get back into work and participate in social life. These can be protective factors respective to the development of psychological disorders [37]. Patients with a higher ECOG state often suffer from severe physical problems beside the impairment through the therapy of HNC. This may additionally contribute to reduced QoL.

Concerning the patient’s weight, we found that patients with lower BMI had significantly worse values in PHQ-9, fatigue and QoL scales. Patients with a lower BMI often have nutritional problems due to pain, swallowing impairment, presence of feeding tube or reduced appetite. Van Liew et al. examined the relationship of weight loss and depression and reported that patients with weight loss within 1 month developed changes in their depressive symptoms in the same period [38]. Best values in PHQ-9 and fatigue scales were reached by patients with BMI 25 to 30. The best QoL was achieved by patients with BMI > 30. Time after diagnosis significantly influenced positively the values in all scales. This could be explained by the disappearing of side effects of the therapy, adaption to the new situation and development of successful coping strategies.

48.3% of our patients had symptoms of depression. In comparison to the general population, suffering from HNC had a high effect on scoring worse values in all scales. Especially in fatigue scale our findings had a high effect size. Concerning the correlation of the single items of all scales Cronbach’s α showed a good or excellent reliability. So, we assessed the used instruments to be appropriate to collect information about the presence of psychological comorbidities of HNC patient in the daily routine practice.

We found the highest positive relationship between depression and anxiety. All scales show an inverse correlation to QoL, distinctly regarding depression and fatigue with a medium correlation of all scales. The finding that depression, fatigue and anxiety negatively influence QoL is consistent to further research [39]. Obviously, patients with additional psychological impairment to their primary disease HNC, have poorer QoL.

### Limitations

A bias cannot surely be excluded by nature of the patient’s selection and the cross-sectional analysis. Sometimes the observed relationships were not even clear concerning the causation of effects. While the selection of the general population groups provided a fair comparison between the HNC group and the general population, other variables such as socioeconomic status might have been different in the groups and could have led to a certain bias. Furthermore, there may be a bias of selection because of distance to therapy. It has to be mentioned that we used one comprehensive questionnaire (PHQ-9), and three short form scales (GAD-2, fatigue and QoL). The tool has to be understood as a screening tool, to find out patient, who need further diagnosis or therapy in this direction. Anyway, the questionnaires were consistent as well as reliable. The large size of the researched sample enabled the detection of small effects.

## Conclusion

The present study shows that there are several factors, which influence psychological side effects in HNC patients. We know from previous studies that early detection of symptoms and unmet needs can improve treatment and outcome of cancer patients [40, 41]. Moreover, pretherapeutic psychological comorbidities have a negative effect on the presence of depressive and fatigue symptoms, as well as they cause impaired survival and functional outcome after diagnosis and therapy [42, 43]. Knowing the impact of age, gender, sociodemographic and clinical factors can sensitize the physician to detect patient’s problems, to share decisions and to develop individual therapy strategies. Perhaps a regular assessment using PRO could help to better identify patients’ risk of developing psychological symptoms.

## Data Availability

The data that support the findings of this study are available on request from the corresponding author VZ. The data are not publicly available due to them containing information that could compromise research participant privacy.

## Abbreviations

HNC: Head and neck cancer
QoL: Quality of life
PRO: Patient reported outcome
ePROM: Electronic patient reported outcome measurement
ICF: International Classification of Functioning
PHQ-9: Patient Health Questionaire - 9
GAD-2: Generalized Anxiety Disorder Questionnaire - 2
EORTC: European Organisation for Research and Treatment of Cancer
ECOG: Eastern Cooperative Oncology Group
BMI: Body Mass Index
ANOVA: Analyses of variance
CFA: Confirmatory factor analysis
TLI: Tucker-Lewis index
RMSEA: Root mean square error of approximation
SRMR: Standardized root mean square residual
HPV: Human papilloma virus
